# Identification of key miRNAs and mRNAs related to coronary artery disease by meta-analysis

**DOI:** 10.1186/s12872-021-02211-2

**Published:** 2021-09-16

**Authors:** Long Liu, Jingze Zhang, Mei Wu, Haiming Xu

**Affiliations:** 1 Department of Cardiovascular Medicine, The Third Hospital of Jilin University, No.126, Xiantai Street, Changchun, 130033 China; 2 Jilin Provincial Precision Medicine Key Laboratory for Cardiovascular Genetic Diagnosis, Changchun, 130033 China; 3 Department of Neurosurgery, The Second Hospital of Jilin University, ChangchunJilin, 130000 China; 4 Human Resources Department, The Third Hospital of Jilin University, Changchun, 130033 China

**Keywords:** Coronary artery disease, Differentially expressed miRNA, Differentially expressed mRNA, Weighted gene co-expression network analysis

## Abstract

**Background:**

To illustrate the mechanism of miRNA and mRNA in coronary artery diseasen (CAD), differentially expressed microRNAs (DEmiRNAs) and genes (DEGs) were analyzed.

**Methods:**

The mRNA transcription profiles of GSE20680 (including 87 blood samples of CAD and 52 blood samples of control), GSE20681 (including 99 blood samples of CAD and 99 blood samples of control) and GSE12288 (including 110 blood samples of CAD and 112 blood samples of control) and the miRNA transcription profiles of GSE59421 (including 33 blood samples of CAD and 37 blood samples of control), GSE49823 (including 12 blood samples of CAD and 12 blood samples of control) and GSE28858 (including 13 blood samples of CAD and 13 blood samples of control) were downloaded from Gene Expression Omnibus (GEO; http://www.ncbi.nlm.nih.gov/geo/). Then, the homogenous expressed mRNAs and miRNAs across the three mRNA transcription profiles and three miRNA transcription profiles were screened using the Fishers exact test in MetaDE. ES package. The weighted gene co-expression network analysis (WGCNA) was used to analyze gene modules. Additionally, the integrated miRNAs–targets regulatory network using the DEmiRNA and their targets was constructed using Cytoscape.

**Results:**

A total of 1201 homogenously statistically significant DEGs were identified including 879 up-regulated and 322 down-regulated DEGs, while a total of 47 homogenously statistically significant DEmiRNAs including 37 up-regulated and 10 down-regulated DEmiRNAs in CAD compared with the controls across the three mRNA transcription profiles and the three miRNA transcription profiles. A total of 5067 genes were clustered into 9 modules in the training dataset, among which, 8 modules were validated. In the miRNAs-targets network, there existed 267 interaction relationships among 5 miRNAs (hsa-miR-361-5p, hsa-miR-139-5p, hsa-miR-146b-5p, hsa-miR-502-5p and hsa-miR-501-5p) and 213 targets. CAV1 could be the target of hsa-miR-361-5 while HSF2 was the target of both hsa-miR-361-5p and hsa-miR-146b-5p. CAV1 was significantly enriched in the GO term of regulation of cell proliferation.

**Conclusion:**

hsa-miR-361-5p, has-miR-146b-5p, CAV1 and HSF2 could play an important role in CAD.

**Supplementary Information:**

The online version contains supplementary material available at 10.1186/s12872-021-02211-2.

## Background

Coronary artery disease (CAD) as the most common type of cardiovascular disease, can lead to a complete blockage of blood flowing to the heart, resulting in a heart attack. For its high morbidity and mortality rate, CAD has probably the most serious cardiovascular disorder threatening people’s health, worldwidely [1].

MicroRNAs (miRNAs) with the length from 18 to 25 nucleotides, is a class of non-protein coding RNA, which has been shown to be involved in a wide variety of biological processes through suppressing the mRNA expression or translation [2]. MiRNAs have been found to express in various tissues and cell types and play important roles in physiological processes including cell growth, proliferation, differentiation, apoptosis, metabolism, and homeostasis as biological regulators [3]. MiRNAs were mostly found in body fluids such as blood and the circulating miRNA expression profiles have been shown to differ significantly between healthy and disease including cancers/tumor, diabetes, neurodegenerative diseases, cardiovascular diseases [3, 4]. So, the blood as one of the most popular non-invasive samples was widely used for identifying potential diagnostic and prognostic biomarkers in human diseases. For CAD, numerous miRNAs have been reported to be circulating biomarkers in the diagnosis of CAD and their functional role in cardiovascular primary prevention has been suggested [5]. For example, the expression levels of miR-126, miR-17, miR-92a, and the inflammation-associated miR-155 in plasma were significantly reduced in patients with CAD compared with healthy controls. [6]. The serum miR-197 and miR-223 could be used to predict cardiovascular death in a cohort of patients with symptomatic CAD [7]. The blood miRNA-19a has been identified as a potential novel biomarker for diagnosis of acute myocardial infarction [8]. However, the miRNAs identified in a single dataset may be limited.

In our study, using three mRNA microarray data of GSE20680 [9], GSE20681 [10] and GSE12288 [10], as well as three miRNA microarray data of GSE59421 [11], GSE49823 [12] and GSE28858 [13], we aimed to further screen the potential biomarkers related to CAD with different analysis methods.

## Materials and methods

### RNA sequencing data

Since our goal was to screen the miRNA and mRNA related to CAD, the key words of coronary artery disease was used to search the Gene Expression Omnibus (GEO; http://www.ncbi.nlm.nih.gov/geo/). As a result, the mRNA transcription profiles of GSE20680 (platform: GPL4133, including 87 blood samples of CAD and 52 blood samples of control), GSE20681 (platform: GPL4133, including 99 blood samples of CAD and 99 blood samples of control) and GSE12288 (platform: GPL4133, including 110 blood samples of CAD and 112 blood samples of control) were downloaded from the GEO. Additionally, the miRNA transcription profiles of GSE59421 (platform: GPL10850, including 33 blood samples of CAD and 37 blood samples of control), GSE49823 (platform: GPL15467, including 12 blood samples of CAD and 12 blood samples of control) and GSE28858 (platform: GPL8179, including 13 blood samples of CAD and 13 blood samples of control) were downloaded from GEO. All the data were downloaded on January 16th, 2020 (Table 1).

**Table 1:** The mRNA and miRNA data information

Accession	Platform	Sample number	CAD(Age(mean ± SD), Gender(Male/Female))	Control(Age(mean ± SD), Gender(Male/Female))
*mRNA*				
GSE20680	GPL4133	139	87 (−,−)	52 (−,−)
GSE20681	GPL4133	198	99 (−,75/24)	99 (−,75/24)
GSE12288	GPL13607	222	110 (54.60 ± 7.11,88/22)	112 (51.94 ± 7.20,84/28)
GSE59421	GPL10850	70	33 (51.18 ± 4.54, −)	37 (51.19 ± 4.54, −)
*miRNA*				
GSE49823	GPL15467	24	12 (60.54 ± 7.98,7/6)	12 (55.77 ± 8.72,5/8)
GSE28858	GPL8179	26	13 (45.67 ± 6.53,13/0)	13 (45.42 ± 6.96,13/0)

### Meta-analysis for differentially expressed RNAs

In order to obtain more reliable miRNAs and mRNAs associated with CAD, a meta-analysis of the microarray datasets was performed.

The homogenous expressed mRNAs and miRNAs across the three mRNA transcription profiles and the three miRNA transcription profiles were screened using the MetaDE. ES algorithm of MetaDE package in R with the thresholds of homogeneity were set as tau2 = 0 and Qpval > 0.05 [14–16]. Subsequently, the homogenously statistically significant differentially expressed genes (DEGs) and differentially expressed miRNAs (DEmiRNAs) were screened with the threshold of false discovery rate (FDR) of < 0.05 using Fishers exact test in metaDE. Moreover, the Fold Change of the DEGs and DEmiRNAs must be simultaneously > 1 or < 1 in each of three mRNA transcription profiles and the three miRNA transcription profiles. The heatmap.sig.genes in MetaDE was used to conduct the bidirectional hierarchical clustering.

### CAD related genes and gene modules screening by WGCNA

WGCNA (weighted gene co-expression network analysis, version 1.61, https://cran.r-project.org/web/packages/WGCNA/index.html) package in R language was used to analyze gene modules and genes that were highly correlated with CAD [15]. The GSE12288 was used as the training dataset to conduct the WGCNA while the GSE20680 and GSE20681 were used as the validation dataset. The power value when the correlation coefficient squared value of connection degree k and p(k) for the first time to reach 0.9 were selected, that was, power = 14; under this power parameter, the mean connectivity degree of the geness was 1, which conformed to the small-world property in a scale-free network. The cutHeight = 0.99 and the gene number ≥ 100 within a module were used as the threshold. The gene moludes with Z-score > 5 and *p* ≤ 0.05 were refered as the preserved gene modules.

### miRNA-mRNA regulatory network construction

Firstly, the targets of the homogenously statistically significant DEmiRNAs were predicted using starBase database (Version 2.0, http://starbase.sysu.edu.cn/). And the targets which were the the homogenously statistically significant DEGs were retained. Secondly, the miRNA-mRNA pairs with the negative correlation were retained. Thirdly, the targets belonging to the gene modules were highlighted. Lastly, the screened miRNA-mRNA pairs were visualized using Cytoscape software (Version 3.6.1, http://www.cytoscape.org) [17]. DAVID (version 6.8, https://david.ncifcrf.gov/) was used to perform the GO and KEGG pathway enrichment for the mRNA in the network [18, 19].

## Results

**Fig. 1:**
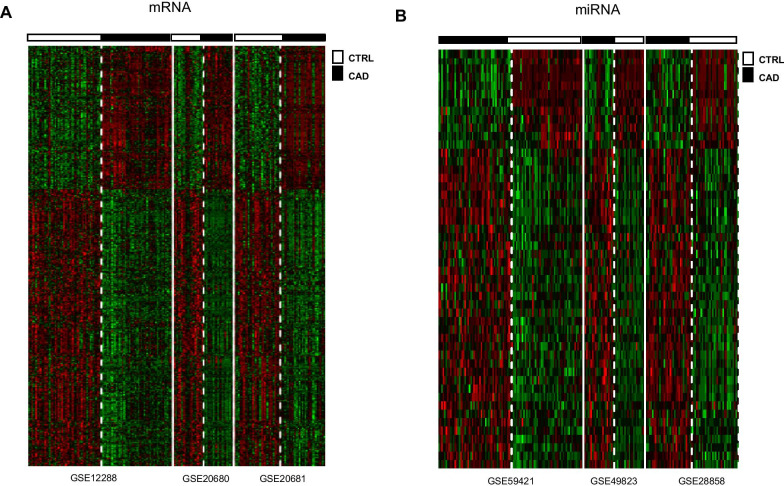
The two-way clustering of differentially expressed RNAs (DErs) between the controls and the coronary artery diseas. **a** For the mRNA; **b** for the miRNAs; up horizontal axis, the samples; vertical axis, datasets; the color from red to green indicated the changes in expression level from high to low

A total of 1201 homogenously statistically significant DEGs were identified including 879 up-regulated and 322 down-regulated DEGs. Additionally, a total of 47 homogenously statistically significant DEmiRNAs including 37 up-regulated and 10 down-regulated DEmiRNAs were screened. The bidirectional hierarchical clustering revealed that DEGs and DEmiRNAs could distinguish the CADs from the controls very well (Fig. 1).

### Modules and genes screened by WGCNA

According to the method, 5067 highly correlated genes were clustered into 9 modules (black, blue, brown, grey, green, red, pink, turquoise and yellow) in the training dataset GSE12288. Among which, 8 modules including modules black, blue, brown, green, red, pink, turquoise and yellow were validated using the validation datasets (Table 2). The Gene dendrogram of the training and validation datasets was shown in Additional file 1: Fig. S1.

**Table 2:** Gene module validation statistics from GSE12288, GSE20681 and GSE20680

ID	Color	Module size	Preservation infor	
			Z-score	*P* value
Module 1	Black	132	15.5129	2.70E − 13
Module 2	Blue	365	29.9215	6.40E − 09
Module 3	Brown	305	10.8946	4.80E − 19
Module 4	Green	222	22.7228	7.00E − 10
Module 5	Grey	2541	3.8535	2.70E − 01
Module 6	Pink	123	5.3986	3.60E − 14
Module 7	Red	152	7.4025	6.00E − 14
Module 8	Turquoise	956	5.4582	1.00E − 20
Module 9	Yellow	271	7.3967	2.10E − 12

Z-score represented the stability of the modules in the two datasets, Z-score > 5 means stable

### MiRNA-mRNA network construction

**Fig. 2:**
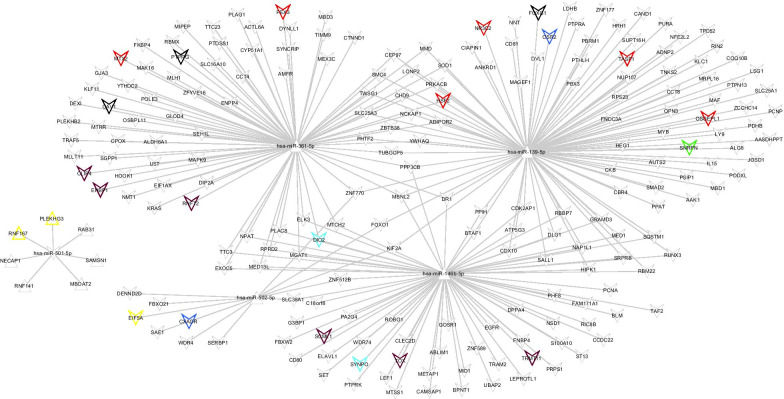
The miRNA-targets regulation network. The triangle nodes represented the up-regulated miRNAs and mRNAs; the quadrangle nodes represented the down-regulated miRNAs and mRNAs; The nodes with colors represented that the targets belonging to the gene modules

In the miRNAs-targets network, there existed 267 interaction relationships among 5 miRNAs (hsa-miR-361-5p, hsa-miR-139-5p, hsa-miR-146b-5p, hsa-miR-502-5p and hsa-miR-501-5p) and 213 targets (Fig. 2). Among the which, CAV1 could be the target of hsa-miR-361-5 while HSF2 was the target of both hsa-miR-361-5p and hsa-miR-146b-5p.

**Fig. 3:**
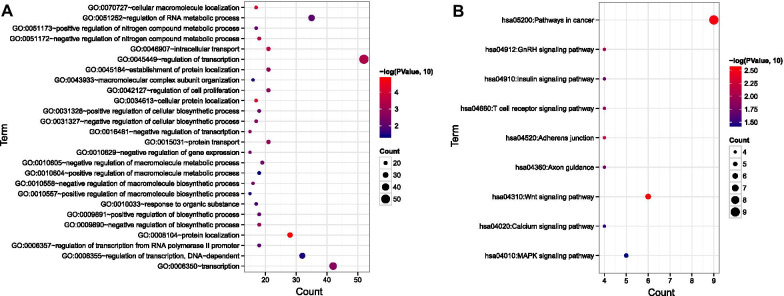
Enriched functions of the targets in the miRNA-mRNA regulation network. **a** For KEGG pathway; **b** for GO terms; Gene numbers are displayed on the x-axis. The color represents the − log (FDR)

Functional enrichment analysis showed that the targets were significantly enriched in 16 GO terms, such as regulation of cell preliferation and 9 KEGG pathways (Fig. 3).

## Discussion

In this study, by using three miRNA microarray and three mRNA microarray of blood samples of CAD and control, a total of 1201 homogenously statistically significant DEGs including 879 up-regulated and 322 down-regulated DEGs and 47 homogenously statistically significant DEmiRNAs including 37 up-regulated and 10 down-regulated DEmiRNAs were identified. Additionally, the WGCNA, miRNA-mRNA regulatory network, functionally enrichment were used to identify the key miRNAs and mRNAs related to CAD. Finally, 5 miRNAs including hsa-miR-361-5p, hsa-miR-139-5p, hsa-miR-146b-5p, hsa-miR-502-5p and hsa-miR-501-5p were identified.

According to the analysis, we found that hsa-miR-146b-5p was significantly up-regulated in the CAD patients. It has been found that miR-146a/b could be involved in CAD by regulating the TLR4 downstream molecules IRAK1 (interleukin-1-receptor-associated kinase 1) and TRAF6 (tumour-necrosis-factor-receptor-associated factor 6) and its level was significantly higher in the CAD group than in the non-CAD group (all *P* < 0.01) [20]. Additionally, miR-146b-5p has also been reported to promote the proliferation, migration and the phenotype transition of vascular smooth muscle cells (VSMCs), which played pivotal roles in vascular remodeling in atherosclerosis [21]. Dysregulation of miR-146a-5p/RHOJ and miR-146b-5p/RHOJ axis in the plasma and ECFCs of CAD patients could be used as biomarkers or therapeutic targets for CAD and other angiogenesis-related diseases [22]. As a result, has -miR-146b-5p could be the potential factor in CAD.

For hsa-miR-361-5p, it was also significantly up-regulated in the CAD patients. Former study has demonstrated the dysregulation of miR-361-5p/VEGF Axis in the plasma and endothelial progenitor cells of patients with CAD [23]. LncRNA MEG3-derived miR-361-5p could regulate VSMCs proliferation and apoptosis by targeting ABCA1 [24]. In the miRNA-mRNA network, hsa-miR-361-5p could regulate the expression of CAV1. The functinal enrichent showed that CAV1 was significantly enriched in the GO term of regulation of cell proliferation. CAV1 which encodes caveolin-1 expressed in cell types relevant to atherosclerosis, was found to be associated with significant risk of CAD when its aberrant expression [25]. So, CAV1 targeted by hsa-miR-361-5p could involved in the mechanism of CAD through the regulation of VSMCs proliferation.

As for hsa-miR-139-5p, hsa-miR-502-5p and hsa-miR-501-5p, there was no study reporting their directed relationship to CAD. However, it has been found that aberrant expression of hsa-miR-139-5p could lead to apoptosis [26] which has been observed in coronary atherosclerosis [27]. hsa-miR-502-5p has been reported to inhibit autophage [28] and the alteration of autophagic genes has also be discovered in CAD [29]. So, these three miRNAs could play an important role in CAD.

In our study, we also found that HSF2 was significantly down-regulated in CAD and was the target of both hsa-miR-361-5p and hsa-miR-146b-5p. HSF2, as a heat shock transcription factors, is more prominently activated during mouse heart development [30]. Former study has demonstrated that HSF2 could induce cardiac hypertrophy during hypertension-induced heart failure by the activation of IGF-IIR [31]. So, we speculated that HSF2 targeted by hsa-miR-361-5p and miR-146b-5p could be related to CAD.

The study’s limitations should be noted. On the one hand, the methods for screening the biomarkers was based on the statistical method rather than the biological experiment. On the other hand, the miRNA and mRNA data were not from the same project. So the additional experiment such as real-time quantitative PCR was needed to validate the miRNA and mRNA expression levels of the same project.

## Conclusions

Above all, we speculated that hsa-miR-361-5p, has-miR-146b-5p, CAV1 and HSF2 could play an important role in CAD. However, further research is required to validate the results.

## Supplementary Information

**Additional file 1** The Gene dendrogram of the training and validation datasets.

## Data Availability

The raw data were collected and analyzed by the Authors, and are not ready to share their data because the data have not been published.

## Abbreviations

DEmiRNAs: Differentially expressed microRNAs
CAD: Coronary artery disease
miRNAs: MicroRNAs
WGCNA: Weighted gene co-expression network analysis
VSMCs: Vascular smooth muscle cells
