# Supplementary material for: Identification of key miRNAs and mRNAs related to coronary artery disease by meta-analysis
Source: BMC Cardiovasc Disord. 2021 Sep 16;21:443. doi: 10.1186/s12872-021-02211-2 (PMC8447760; doi:10.1186/s12872-021-02211-2)
Supplement: Supplementary file 1 — Additional file 1 The Gene dendrogram of the training and validation datasets. [file 12872_2021_2211_MOESM1_ESM.pdf]

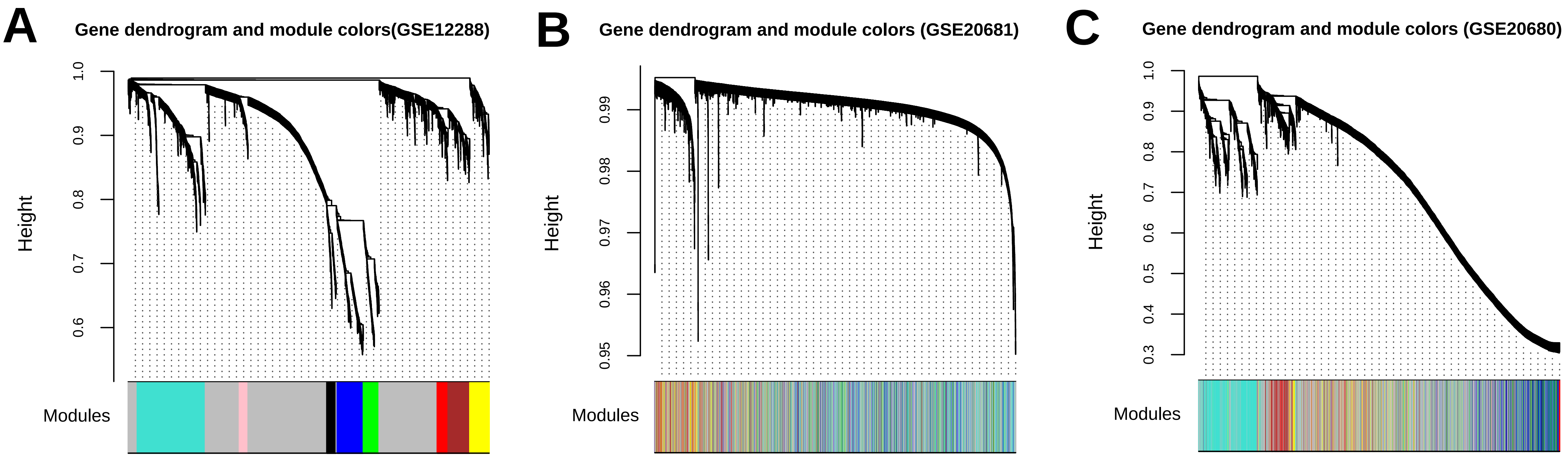
A
Gene dendrogram and module colors(GSE12288)
Height
1.0
0.9
0.8
0.7
0.6
Modules
B
Gene dendrogram and module colors (GSE20681)
Height
0.99
0.98
0.97
0.96
0.95
Modules
C
Gene dendrogram and module colors (GSE20680)
Height
1.0
0.9
0.8
0.7
0.6
0.5
0.4
0.3
Modules
